# Novel Pathogenic Variants in *PJVK*, the Gene Encoding Pejvakin, in Subjects with Autosomal Recessive Non-Syndromic Hearing Impairment and Auditory Neuropathy Spectrum Disorder

**DOI:** 10.3390/genes13010149

**Published:** 2022-01-15

**Authors:** María Domínguez-Ruiz, Montserrat Rodríguez-Ballesteros, Marta Gandía, Elena Gómez-Rosas, Manuela Villamar, Pietro Scimemi, Patrizia Mancini, Nanna D. Rendtorff, Miguel A. Moreno-Pelayo, Lisbeth Tranebjaerg, Carme Medà, Rosamaria Santarelli, Ignacio del Castillo

**Affiliations:** 1 Servicio de Genética, Hospital Universitario Ramón y Cajal, IRYCIS, 28034 Madrid, Spain; mdominguezr@salud.madrid.org (M.D.-R.); montserodrig@yahoo.es (M.R.-B.); martagandia04@gmail.com (M.G.); elenagomez_tel@yahoo.es (E.G.-R.); taugen.hrc@salud.madrid.org (M.V.); mmorenop@salud.madrid.org (M.A.M.-P.); 2 Centro de Investigación Biomédica en Red de Enfermedades Raras (CIBERER), 28034 Madrid, Spain; 3 Department of Neurosciences, University of Padua, 35121 Padua, Italy; pietro.scimemi@unipd.it (P.S.); rosamaria.santarelli@unipd.it (R.S.); 4 Audiology Service, Santi Giovanni e Paolo Hospital, 30122 Venice, Italy; 5 Department of Sense Organs, University La Sapienza, 00162 Rome, Italy; p.mancini@uniroma1.it; 6 Department of Clinical Genetics, University Hospital, Copenhagen/The Kennedy Centre, DK-2600 Glostrup, Denmark; nanna.dahl.rendtorff@regionh.dk (N.D.R.); tranebjaerg@sund.ku.dk (L.T.); 7 Department of Clinical Medicine, University of Copenhagen, DK-2100 Copenhagen, Denmark; 8 Unidad de Prevención de Enfermedades del Oído, Conselleria de Salut, Illes Balears, 07120 Palma de Mallorca, Spain; cmeda@dgsanita.caib.es

**Keywords:** non-syndromic hearing impairment, auditory neuropathy spectrum disorder, DFNB59, *PJVK*, pejvakin, genetic epidemiology

## Abstract

Pathogenic variants in the *PJVK* gene cause the DFNB59 type of autosomal recessive non-syndromic hearing impairment (AR-NSHI). Phenotypes are not homogeneous, as a few subjects show auditory neuropathy spectrum disorder (ANSD), while others show cochlear hearing loss. The numbers of reported cases and pathogenic variants are still small to establish accurate genotype-phenotype correlations. We investigated a cohort of 77 Spanish familial cases of AR-NSHI, in whom DFNB1 had been excluded, and a cohort of 84 simplex cases with isolated ANSD in whom *OTOF* variants had been excluded. All seven exons and exon-intron boundaries of the *PJVK* gene were sequenced. We report three novel DFNB59 cases, one from the AR-NSHI cohort and two from the ANSD cohort, with stable, severe to profound NSHI. Two of the subjects received unilateral cochlear implantation, with apparent good outcomes. Our study expands the spectrum of *PJVK* mutations, as we report four novel pathogenic variants: p.Leu224Arg, p.His294Ilefs*43, p.His294Asp and p.Phe317Serfs*20. We review the reported cases of DFNB59, summarize the clinical features of this rare subtype of AR-NSHI and discuss the involvement of *PJVK* in ANSD.

## 1. Introduction

Inherited hearing impairment is clinically and genetically very heterogeneous. Hearing loss can be an isolated condition (non-syndromic hearing impairment, NSHI) or it can be part of the clinical signs that are characteristic of specific genetic syndromes [1]. Over 120 genes are currently known to be involved in NSHI, and it is estimated that many more remain to be identified [2]. For most of the known genes, few affected subjects have been reported to carry causative variants, and this poor knowledge of the mutational spectra is hindering the investigation of genotype-phenotype correlations in the different genetic types of NSHI [3].

The DFNB59 type of autosomal recessive (AR) NSHI (MIM #610220) is caused by pathogenic variants in the *PJVK* gene (MIM #610219) [4], which is located on 2q31.2, spanning 9950 bp of genomic sequence. It contains seven exons and codes for pejvakin, a 352-residue protein that belongs to the gasdermin family. Six different gasdermins are known in humans (gasdermins A to E, and pejvakin) [5]. The five canonical members of the family (gasdermins A-E) contain an N-terminal membrane-permeabilizing domain, a short linker region, and a C-terminal autoinhibitory domain. Proinflammatory signals result in the separation of the two domains through caspase-mediated cleavage at the linker region, so that the N-terminal domain is released and can form pores in the plasma membrane. Depending on which gasdermin is activated, this mechanism triggers different types of programmed cell death (pyroptosis, secondary necrosis or NETosis) [6]. In contrast, pejvakin is a non-canonical gasdermin, as it lacks the cleavable linker and the C-terminal autoinhibitory domain, which is substituted by a zinc-finger domain whose function is unknown. Pejvakin has been reported to localize to the stereociliary rootlets of the inner ear hair cells, where it would be needed for stereocilia maintenance [7]. In other studies, pejvakin has been reported to be associated with peroxisomes, where it would mediate their autophagic degradation (pexophagy) as a protective mechanism against the oxidative stress that is caused by noise overexposure [8,9].

Up to 19 different variants in *PJVK* have been reported as causative of AR-NSHI in families from diverse geographic origins [4,10,11,12,13,14,15,16,17,18,19,20,21,22,23,24,25,26]. In a study performed on four Iranian families, three of them carrying the same homozygous variant (c.547C>T, p.Arg183Trp), the hearing impairment showed features of auditory neuropathy spectrum disorder (ANSD), i.e., abnormal or absent auditory brainstem responses but normal otoacoustic emissions [4]. This clinical feature is in accordance with the phenotype observed in a knock-in mouse model for the p.Arg183Trp variant [4]. However, ANSD was not observed in any of the few other reported DFNB59 cases in whom this condition was tested, nor in the *sirtaki* mouse, which was obtained by ENU mutagenesis and carries a nonsense variant in *Pjvk* [13]. Clarification of this controversial issue needs a better knowledge of the *PJVK* variant spectrum and the resulting phenotypes, through the investigation of large cohorts of hearing-impaired subjects, with or without ANSD.

In this study, we have screened a cohort of 77 familial cases of non-DFNB1 AR-NSHI, and a cohort of 84 subjects with isolated ANSD. We report the first European cases of DFNB59 NSHI. Five *PJVK* pathogenic variants, four of them novel, were found in three unrelated cases whose clinical characterization further illustrates the phenotypic variability of the *PJVK* type of hearing impairment.

## 2. Materials and Methods

### 2.1. Human Subjects

Two cohorts of subjects were enrolled in this study. The first cohort consisted of 140 Spanish familial cases of autosomal recessive NSHI (AR-NSHI), with at least two affected siblings and unaffected parents. Prior to this work, they were investigated by Sanger sequencing of the coding region and splice sites of the *GJB2* gene and by testing for the common del(*GJB6*-D13S1830) and del(*GJB6*-D13S1854) deletions, which revealed causative variants in 63 families. The remaining 77 families were investigated for variants in the *PJVK* gene. The second cohort consisted of 84 simplex cases (40 from Spain, 23 from Italy, 21 from Denmark) with isolated AN in whom pathogenic variants in the *OTOF* gene, encoding otoferlin, had been excluded previously. After approval by the Ethical Committee of Hospital Universitario Ramón y Cajal (in accordance with the 1964 Declaration of Helsinki), written informed consent was obtained from all participating subjects.

### 2.2. Clinical Tests

Hearing was evaluated by pure-tone audiometry, testing for air conduction (frequencies 250–8000 Hz) and bone conduction (frequencies 250–4000 Hz). The degree of hearing impairment was defined by the pure tone average (PTA) threshold levels at 0.5, 1, 2 and 4 kHz, and was classified as mild (21–40 dB HL), moderate (41–70 dB HL), severe (71–95 dB HL) and profound (>95 dB HL). ANSD was diagnosed on the basis of absent or grossly abnormal auditory-evoked brainstem responses (ABR) and preserved otoacoustic emissions (OAE) [27]. Speech perception tests were performed on Italian subject E1471 II:1 in the auditory-only listening condition using live-voice presentation. The speech material consisted of disyllabic words obtained from an Italian adaptation [28] of the word lists in the Northwestern University-Children’s Perception of Speech (NU-CHIPs) tool [29].

### 2.3. DNA Purification, Genotyping and Sequencing

DNA was extracted from peripheral blood samples by using the Chemagic MSM I automated system (Chemagen, Baesweiler, Germany). Microsatellite markers D2S148, D2S2173, D2S324 and D2S2310 were amplified using fluorescently-labeled primers and PCR conditions as previously reported [30]. Amplified alleles were resolved by capillary electrophoresis in an ABI Prism 3100 Avant Genetic Analyzer (Applied Biosystems, Waltham, MA, USA). Primers and conditions for PCR amplification of all seven exons of the *PJVK* gene are shown in Table 1. Sanger DNA sequencing was performed in an ABI Prism 3100 Avant Genetic Analyzer (Applied Biosystems, Waltham, MA, USA).

### 2.4. Assessment of Pathogenicity of DNA Variants

Pathogenicity of DNA variants was assessed according to the guidelines from the American College of Medical Genetics and Genomics and the Association for Molecular Pathology (ACMG/AMP) [31], as implemented by Varsome [32], using GRCh38 as human reference genome. Scores were subsequently modified manually to delete criterion PP2 and to take into consideration criterion PM3, as recommended in the disease-specific ACMG/AMP guidelines for hearing loss [33].

## 3. Results

### 3.1. Genetic Study

**Figure 1:**
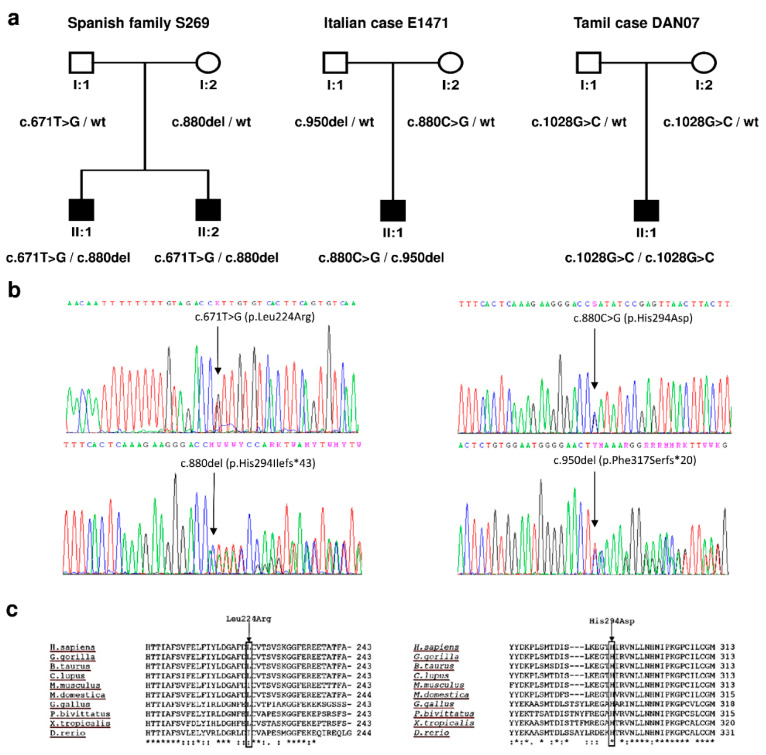
Novel pathogenic variants that were found in this study. (**a**) Pedigrees showing the segregation of variants. (**b**) Electropherograms from subject S269 II:1 (left panel) and from subject E1471 II:1 (right panel). (**c**) Alignment of pejvakin orthologous sequences from human and nine other vertebrates. Asterisks indicate identical residues across all sequences; colons, conserved positions (residues of strongly similar properties); periods, semi-conserved positions (residues of weakly similar properties). Sequence accesion numbers: *Homo sapiens* (NP_001036167.1); *Gorilla gorilla* (XP_004032916.1); *Bos taurus* (NP_001180112.1); *Canis lupus* (XP_535979.2); *Mus musculus* (NP_001074180.1); *Monodelphis domestica* (XP_001368857.1); *Gallus gallus* (XP_426573.2); *Python bivittatus* (XP_007433246.1); *Xenopus tropicalis* (XP_012826511.1); *Danio rerio* (XP_009300492.1).

We investigated a cohort of 77 Spanish familial cases of autosomal recessive non-syndromic hearing loss, with at least two affected siblings, in whom DFNB1 pathogenic variants had been previously excluded. Firstly, all siblings in the family and their parents were genotyped for microsatellite markers D2S148, D2S2173, D2S324 and D2S2310, closely linked to *PJVK*. In 23 families in which haplotype analysis could not exclude genetic linkage, we sequenced all exons and exon/intron boundaries of *PJVK* from one affected sibling. We found likely causative variants in Spanish family S269. The two affected brothers were compound heterozygous for the novel variants c.671T>G (p.Leu224Arg) and c.880del (p.His294Ilefs*43), whereas the father carried c.671T>G, and the mother carried c.880del (Figure 1). The family had no siblings with normal hearing.

We also screened a cohort of 84 simplex cases (40 from Spain, 23 from Italy, 21 from Denmark) with isolated ANSD in whom pathogenic variants in the *OTOF* gene, encoding otoferlin, had been excluded previously. All exons and exon/intron boundaries of the *PJVK* gene were sequenced in every case. We found likely causative variants in two unrelated subjects, who had no siblings with normal hearing. Italian subject E1471-1 was compound heterozygous for the novel variants c.880C>G (p.His294Asp) and c.950del (p.Phe317Serfs*20). His father carried c.950del, and his mother c.880C>G. Subject DAN7-1, from the cohort recruited in Denmark but of Tamil ethnic origin, was homozygous for the previously reported c.1028G>C (p.Cys343Ser) variant. His parents were heterozygous carriers for this variant. Cys-343 has been shown to play a crucial role in the interaction between pejvakin and LC3B, an autophagosomal marker in the pexophagy pathway [9].

Two of the novel variants are single-base deletions that result in frame shifts, leading to truncated polypeptides or mRNA degradation by nonsense-mediated decay. The two other novel variants are missense, which affect evolutionarily conserved residues in the pejvakin polypeptide (Figure 1C). They were classified as deleterious/probably damaging according to the scores provided by SIFT and Polyphen-2 (Table 2). They segregate with the disease as expected from an autosomal recessive pattern, and each variant is in trans with a truncating variant in affected subjects (Figure 1A). They were found at very low frequencies in the Genome Aggregation Database [34]. Both variants were classified as “likely pathogenic” according to the ACMG/AMP guidelines [31,33] (Table 2). Therefore, the reported novel genotypes in cases S269 and E1471 are considered to be causative of the hearing impairment of the affected subjects.

### 3.2. Clinical Study

**Figure 2:**
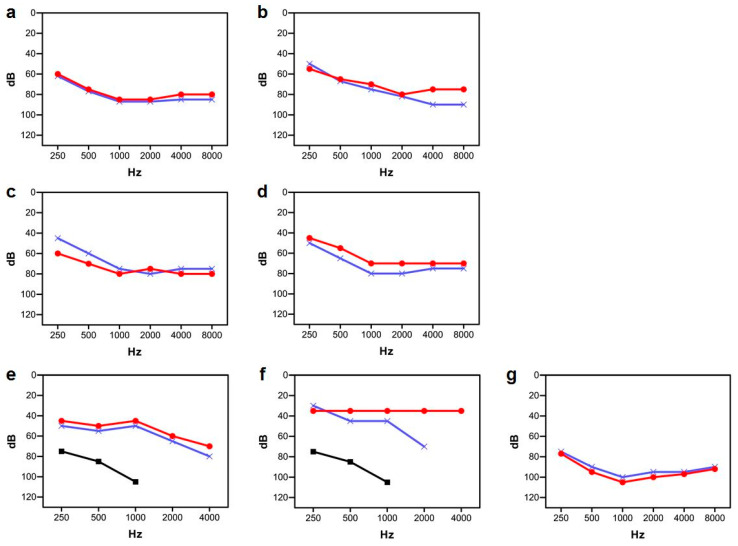
Audiograms from subjects with *PJVK* variants causing sensorineural hearing loss. Only results for air conduction are shown. Red line, right ear. Blue line, left ear. (**a**,**b**) Subject S269 II:1 at ages 7 yr and 14 yr, respectively. (**c**,**d**) Subject S269 II:2 at ages 5 yr and 11 yr, respectively. (**e**) Subject E1471 II:1, while using hearing aids in both ears (red and blue lines); black line, unaided hearing. (**f**) Subject E1471 II:1, while using the cochlear implant in the right ear (red line) and a hearing aid in the left ear (blue line); black line, unaided hearing. (**g**) Subject DAN07 II:1 at age 12 yr, before unilateral cochlear implantation.

In Spanish family S269, the two affected brothers had not been subjected to newborn hearing screening. Subject II:1 was diagnosed with non-syndromic hearing impairment by age four years. Because of this familial history, his brother (II:2) received a similar diagnosis earlier, at age two years. Both presented with a severe hearing loss, which seems to be stable, as shown by serial pure-tone audiograms along 10 years of evolution (Figure 2a–d). Their parents had normal hearing. The two brothers were tested for otoacoustic emissions at ages six and three years, respectively, with no response bilaterally. ABR recordings were performed at these same ages, and the results were consistent with a severe hearing impairment. Computed Tomography (CT) scan of subject II:1 did not reveal any abnormal findings.

In Italian case E1471, subject II:1 had normal growth and motor development, and he showed no risk factors for hearing loss. He had not been subjected to newborn hearing screening and was referred for assessment because of parental concern regarding his hearing at the age of two years. ABR recordings showed no response at the maximum stimulation intensity (90 dB nHL) while OAE were detected in both ears and disappeared thereafter. Behavioral reinforced audiometry, performed at the age of four years, indicated profound hearing loss. Pure tone average (PTA) threshold at 0.5, 1, 2 and 4 kHz measured in the free field was higher than 110 dB HL. The child was first fitted with power hearing aids, which resulted in a considerable improvement in pure tone sensitivity (Figure 2e); however, the aided thresholds were above the range of conversational speech and there was a considerable delay in development of language skills. Both CT and magnetic resonance imaging (MRI) head and ear (including internal acoustic canal) scans were normal.

At age five years, the child received a cochlear implant (MED-EL Synchrony, MED-EL, Innsbruck, Austria) in the right ear. Electrically-evoked auditory nerve responses (electrically-evoked compound action potentials, e-CAPs) were recorded through the cochlear implant. The aided thresholds, measured in the free field with the child wearing the cochlear implant (Figure 2f), fell within the estimated range of estimated conversational speech [35]. Disyllable recognition scores improved from the pre-implant value of 20% to 90% within one year of cochlear implant use.

At age nine years, the child was using the cochlear implant in the right ear and a hearing aid in the other ear. Scores of the speech perception tests were as follows: recognition of disyllabic words, in bimodal configuration and quiet environment: 85%; recognition of sentences, in bimodal configuration and quiet environment: 90%; recognition of disyllabic words, with cochlear implant only, in quiet environment: 70%. Overall, speech perception is satisfactory. In contrast, language is poorly developed for the chronological age. Indeed, scores on lexical comprehension and production would be adequate only for the age of five years (standardized tests in Italian language: Peabody, Rustioni).

Case DAN07 was recruited in Denmark, but the family is of Tamil origin. Subject II:1 was diagnosed with hearing impairment at age six months. Both CT and MRI scans were normal. ABR recordings and pure-tone audiometry revealed a profound hearing loss (Figure 2g). Electrocochleography records were abnormal and compatible with auditory neuropathy. He was initially treated with hearing aids. At age 12 years, he received a cochlear implant in the right ear, with apparent good outcomes in aided hearing thresholds, but a careful follow-up is needed to confirm this conclusion.

## 4. Discussion

Here we report the first European cases of DFNB59 hearing impairment, including four novel pathogenic variants that expand the mutational spectrum of *PJVK*. Both the Spanish and Italian cases had only Spanish and Italian ancestors, respectively, beyond at least three generations, which supports their European origins. Taking into account the four novel variants, 23 pathogenic variants have hitherto been reported in this gene (Table 3), all of them in cases of AR-NSHI. The list includes 15 truncating variants, seven missense variants and one in-frame deletion of a single codon. Most of the variants have been reported in the homozygous state in cases from Iran, Pakistan and Turkey (Table 4). In European populations, pathogenic *PJVK* variants seem to be a rare cause of, as observed in our study (1 case out of 140 families with AR-NSHI, i.e., 0.7%) and in previous works, which did not find any DFNB59 case in a series of cohorts from different European countries (reviewed in [36]).

Although clinical data in the literature are far from being complete (Table 4), it is possible to start delineating some clinical features of the DFNB59 AR-NSHI. Onset is prelingual in a great majority of cases, and subjects with the onset reported in early childhood are likely to reflect a delay in diagnosis because they were not subjected to newborn hearing screening (e.g., cases S269 and E1471 of this study). The evolution of the hearing impairment has been reported to be stable or progressive in equal proportions (Table 4). In most cases, the hearing loss ranges from severe to profound (Table 4). In one of the two cases in whom it is moderate, it progressed to profound over the years [13]. None of these features is associated to any specific combination of truncating or non-truncating variants.

ANSD was postulated to be a clinical feature of DFNB59 on the basis of the study of four Iranian families, in the first report of *PJVK* variants as a cause of AR-NSHI [4]. In three of the families, affected subjects were homozygous for p.Arg183Trp, and in the other one, they were homozygous for p.Thr54Ile. In 11 of 12 affected subjects (four with p.Arg183Trp, and eight with p.Thr54Ile), normal synchronized spontaneous OAE (SSOAE) were recorded (ages of subjects at testing, 12–23 years). In contrast, ABR were absent or showed thresholds higher than 80 dB in all subjects. Unfortunately, ANSD was not specifically investigated in most of the DFNB59 cases that were reported subsequently (Table 4). However, in 10 of the 12 remaining cases who were tested (one of them, case S269 in this study), normal OAE could not be recorded. In case E1471 (this work), OAE were recorded at an early age and they disappeared thereafter. Of note, all cases with a diagnosis of ANSD share the feature of carrying at least one allele with a missense variant [4], this work. This would suggest that ANSD could be associated with specific non-truncating variants. However, cases without ANSD have been reported with the same genotypes (p.Arg183Trp or p.Cys343Ser in the homozygous state) as cases with ANSD.

Murine models do not shed light on this issue, as they reproduce the situation that has been observed in humans. A knock-in mouse model for the p.Arg183Trp variant in *PJVK* shows ANSD [4]. However, OAE records were abnormal in the *sirtaki* mouse, which carries a nonsense variant in *Pjvk* [13], and in *Pjvk*-null mice carrying a deletion of whole exon 2 [8]. Moreover, the expression and function of pejvakin in the inner ear and auditory pathway still need clarification. Expression of pejvakin was reported in the hair cells of the organ of Corti, in the spiral ganglion neurons, and in the first three relays of the afferent auditory pathway (cell bodies of neurons from the cochlear nuclei, superior olivary complex and inferior colliculus) [4,13]. However, selective ablation of murine *Pjvk* in spiral ganglion neurons did not result in hearing impairment [7]. As regards pejvakin function, two different roles have been postulated. Pejvakin would be needed for stereocilia maintenance in hair cells, by interacting with proteins of the stereociliary rootlets [7]. Pejvakin has also been reported to mediate pexophagy, the autophagic degradation of peroxisomes, as a protective mechanism against the oxidative stress that is caused by noise overexposure [8,9]. Accordingly, the lack of this protective mechanism may explain the progressive hearing impairment that is observed in some DFNB59 patients. If the primary lesion in DFNB59 patients occurred in the hair cells, it could be hypothesized that inner hair cells would be affected earlier than outer hair cells in some subjects [7]. Consequently, ABR would be abnormal whereas OAE could be recorded during a short period of time, as in case E1471. If tested later, those DFNB59 patients would not be diagnosed with ANSD.

On the basis of the expression of pejvakin in the auditory pathway, it was hypothesized that the outcomes of cochlear implants in DFNB59 patients may not be good. Here we report case E1471, with an early diagnosis of ANSD, who received a cochlear implant in the right ear at the age of five years. Four years later, speech perception tests show good results, but language development is delayed. Although this delay could be related to the relatively late age of implantation, careful follow-up is needed for a correct evaluation of this case. Data on the outcome of cochlear implants in many other DFNB59 patients should be collected before any recommendation may be issued to orientate the choice of therapy in subjects with this subtype of AR-NSHI.

## Figures and Tables

**Table 1:** Primers and conditions for PCR amplification of all exons of *PJVK*.

Exon	Primer Sequences (5′-3′)	[MgCl_2_]
1	F: CTAGGCCGCAGTTCTTTGTCCTTAG R: TCCCAGGCAAACGCCATTACA	2.5 mM
2	F: GCAGAGGCAGGGAATTATACAGT R: ACAAACTTTTGGCATTGTTAATCTT	2.0 mM
3	F: TGGTGAGTCATGTTGCCTTTCT R: CAACCTCAATGTTTTAAGCATTCTT	1.5 mM
4	F: CTGACTATTAGGATTGCCTTGATTT R: CAGCTCTTTCATCAGAACATTTCA	1.5 mM
5	F: TTGTTTTTGGTAGGATTATAGGAAA R: GAGAGCACATGCCCTAATGAAT	2.5 mM
6	F: TCATCACCCCATCAAACAATAA R: GAATAGAAAACCTCATGTGTTAAGC	1.5 mM
7	F: GCTGTTTGCATTATGTATTTTTCA R: TGTGGCACAACTGCACCTAA	2.0 mM

F, forward; R, reverse; annealing temperature of 60 °C for all amplicons.

**Table 2:** Assessment of pathogenicity of the novel missense variants in *PJVK*.

Variant		SIFT Score	Polyphen-2 Score	Minor Allele Frequency (MAF) [31]	ACMG Criteria	Classification
DNA	Protein					
c.671T->G	p.Leu224Arg	0.01 (deleterious)	0.959 (Probably damaging)	2 × 10^−5^ (global) 4 × 10^−5^ (Non-Finnish Europeans)	PM2 (strong), PM3 (strong), PP1 (supporting)	Likely pathogenic
c.880C>G	p.His294Asp	0.00 (deleterious)	0.981 (Probably damaging)	4 × 10^−6^ (global) 8 × 10^−6^ (Non-Finnish Europeans)	PM2 (strong), PM3 (moderate)	Likely pathogenic

**Table 3:** Pathogenic variants reported to date in *PJVK* (NM_001042702.3).

Exon	DNA Level	Protein Level	Reference
2	c.113dup	p.Lys41Glufs*8	[11,26]
2	c.122del	p.Lys41Serfs*18	[13,16]
2	c.147T>A	p.Tyr49*	[23]
2	c.158C>G	p.Ser53*	[24]
2	c.161C>T	p.Thr54Ile	[4]
2	c.162_172del	p.Pro55fs*23	[24]
intron 2	c.211+1G>T		[18]
3	c.274C>T	p.Arg92*	[16,18]
3	c.406C>T	p.Arg136*	[14,15,24,26]
4	c.485G>A	p.Ser162Asn	[25]
4	c.499C>T	p.Arg167*	[12,18,20]
4	c.547C>T	p.Arg183Trp	[4,12,21,22]
6	c.671T>G	p.Leu224Arg	This work
6	c.726del	p.Phe242Leufs*7	[10]
6	deletion of whole exon		[18]
7	c.880del	p.His294Ilefs*43	This work
7	c.880C>G	p.His294Asp	This work
7	c.908_910del	p.Asn303del	[24]
7	c.930_931del	p.Cys312Trpfs*19	[19]
7	c.950del	p.Phe317Serfs*20	This work
7	c.970G>T	p.Gly324Trp	[18]
7	c.988del	p.Val330Leufs*7	[10]
7	c.1028G>C	p.Cys343Ser	[17], This work

**Table 4:** Genotypes and phenotypes observed in subjects with the DFNB59 type of autosomal recessive hearing impairment.

Genotype	Families	Features of the Hearing Loss				Reference
		Onset	Severity	Evolution	AN	
p.[Lys41Glufs*8];[Lys41Glufs*8]	1 (Moroccan)	Prelingual	S-P	Progressive	No	[11]
	1 (Moroccan)	Prelingual	P	NR	NT	[26]
p.[Lys41Serfs*18];[Lys41Serfs*18]	1 (Iranian)	NR	M-P	Progressive	NT	[13]
	1 (Iranian)	Prelingual	P	Progressive	NT	[16]
p.[Tyr49*];[Tyr49*]	1 (Pakistani)	Prelingual	NR	NR	NT	[23]
p.[Ser53*];[Ser53*]	1 (Pakistani)	NR	NR	NR	NT	[24]
p.[Thr54Ile];[Thr54Ile]	1 (Iranian)	Prelingual	S	NR	Yes	[4]
p.[Pro55fs*23];[Pro55fs*23]	1 (Pakistani)	NR	NR	NR	NT	[24]
c.[211 + 1G > T];[211 + 1G > T]	1 (Iranian)	Prelingual	NR	NR	NT	[18]
p.[Arg92*];[Arg92*]	1 (Iranian)	Prelingual	S-P	Stable	NT	[16]
	1 (Iranian)	Prelingual	S	NR	NT	[18]
p.[Arg136*];[Arg136*]	1 (Palestinian)	Prelingual	P	NR	No	[14]
	3 (Israeli Arab)	Prelingual	M-S	Stable	No	[15]
	1 (Pakistani)	NR	NR	NR	NT	[24]
	1 (Moroccan)	Prelingual	P	NR	NT	[26]
p.[Ser162Asn];[p.Ser162Asn]	1 (Pakistani)	Prelingual	P	NR	NT	[25]
p.[Arg167*];[Arg167*]	1 (Turkish)	NR	S-P	NR	No	[12]
	1 (Iranian)	Prelingual	P	NR	NT	[18]
	1 (Turkish)	Prelingual	NR	NR	NT	[20]
p.[Arg183Trp];[Arg183Trp]	3 (Iranian)	Prelingual	P	NR	Yes	[4]
	1 (Turkish)	Prelingual	S-P	NR	No	[12]
	1 (Iranian)	Prelingual	NR	NR	NT	[21]
	1 (Iranian)	NR	NR	NR	NT	[22]
p.[Leu224Arg];[His294Ilefs*43]	1 (Spanish)	Early childhood	S	Stable	No	This work
p.[Phe242Leufs*7];[Phe242Leufs*7]	1 (Iranian)	NR	P	NR	NT	[10]
Homozygous deletion of exon 6	1 (Iranian)	Prelingual	NR	NR	NT	[18]
p.[His294Asp];[Phe317Serfs*20]	1 (Italian)	Early childhood	P	Stable	Yes	This work
p.[Asn303del];[Asn303del]	1 (Pakistani)	NR	NR	NR	NT	[24]
p.[Cys312Trpfs*19];[Cys312Trpfs*19]	1 (Chinese)	Prelingual	S-P	Progressive	No	[19]
p.[Gly324Trp];[Gly324Trp]	1 (Iranian)	Prelingual	S-P	NR	NT	[18]
p.[Val330Leufs*7];[Val330Leufs*7]	1 (Iranian)	NR	P	NR	No	[10]
p.[Cys343Ser];[Cys343Ser]	1 (Pakistani)	Early childhood	S-P	Progressive	NT	[17]
	1 (Tamil)	Prelingual	P	Stable	Yes	This work

AN, auditory neuropathy; M, moderate; S, severe; P, profound; NR, not reported; NT, not tested.

## Data Availability

Data on the novel pathogenic variants that are reported in this study are available in ClinVar (accession numbers SCV002043792 to SCV002043795).

## References

[1] Dror A.A., Avraham K.B. (2010). Hearing impairment: A panoply of genes and functions. Neuron.

[2] Van Camp G., Smith R.J. Hereditary Hearing Loss Homepage. https://hereditaryhearingloss.org.

[3] Hoefsloot L.H., Feenstra I., Kunst H.P., Kremer H. (2014). Genotype phenotype correlations for hearing impairment: Approaches to management. Clin. Genet..

[4] Delmaghani S., del Castillo F.J., Michel V., Leibovici M., Aghaie A., Ron U., Van Laer L., Ben-Tal N., Van Camp G., Weil D. (2006). Mutations in the gene encoding pejvakin, a newly identified protein of the afferent auditory pathway, cause DFNB59 auditory neuropathy. Nat. Genet..

[5] De Schutter E., Roelandt R., Riquet F.B., Van Camp G., Wullaert A., Vandenabeele P. (2021). Punching Holes in Cellular Membranes: Biology and Evolution of Gasdermins. Trends Cell Biol..

[6] Rogers C., Alnemri E.S. (2019). Gasdermins in apoptosis: New players in an old game. Yale J. Biol. Med..

[7] Kazmierczak M., Kazmierczak P., Peng A.W., Harris S.L., Shah P., Puel J.L., Lenoir M., Franco S.J., Schwander M. (2017). Pejvakin, a Candidate Stereociliary Rootlet Protein, Regulates Hair Cell Function in a Cell-Autonomous Manner. J. Neurosci..

[8] Delmaghani S., Defourny J., Aghaie A., Beurg M., Dulon D., Thelen N., Perfettini I., Zelles T., Aller M., Meyer A. (2015). Hypervulnerability to Sound Exposure through Impaired Adaptive Proliferation of Peroxisomes. Cell.

[9] Defourny J., Aghaie A., Perfettini I., Avan P., Delmaghani S., Petit C. (2019). Pejvakin-mediated pexophagy protects auditory hair cells against noise-induced damage. Proc. Natl. Acad. Sci. USA.

[10] Chaleshtori M.H., Simpson M.A., Farrokhi E., Dolati M., Hoghooghi-Rad L., Amani Geshnigani S., Crosby A.H. (2007). Novel mutations in the pejvakin gene are associated with autosomal recessive non-syndromic hearing loss in Iranian families. Clin. Genet..

[11] Ebermann I., Walger M., Scholl H.P., Charbel Issa P., Lüke C., Nürnberg G., Lang-Roth R., Becker C., Nürnberg P., Bolz H.J. (2007). Truncating mutation of the DFNB59 gene causes cochlear hearing impairment and central vestibular dysfunction. Hum. Mutat..

[12] Collin R.W., Kalay E., Oostrik J., Caylan R., Wollnik B., Arslan S., den Hollander A.I., Birinci Y., Lichtner P., Strom T.M. (2007). Involvement of DFNB59 mutations in autosomal recessive nonsyndromic hearing impairment. Hum. Mutat..

[13] Schwander M., Sczaniecka A., Grillet N., Bailey J.S., Avenarius M., Najmabadi H., Steffy B.M., Federe G.C., Lagler E.A., Banan R. (2007). A forward genetics screen in mice identifies recessive deafness traits and reveals that pejvakin is essential for outer hair cell function. J. Neurosci..

[14] Shahin H., Walsh T., Rayyan A.A., Lee M.K., Higgins J., Dickel D., Lewis K., Thompson J., Baker C., Nord A.S. (2010). Five novel loci for inherited hearing loss mapped by SNP-based homozygosity profiles in Palestinian families. Eur. J. Hum. Genet..

[15] Borck G., Rainshtein L., Hellman-Aharony S., Volk A.E., Friedrich K., Taub E., Magal N., Kanaan M., Kubisch C., Shohat M. (2012). High frequency of autosomal-recessive DFNB59 hearing loss in an isolated Arab population in Israel. Clin. Genet..

[16] Babanejad M., Fattahi Z., Bazazzadegan N., Nishimura C., Meyer N., Nikzat N., Sohrabi E., Najmabadi A., Jamali P., Habibi F. (2012). A comprehensive study to determine heterogeneity of autosomal recessive nonsyndromic hearing loss in Iran. Am. J. Med. Genet..

[17] Mujtaba G., Bukhari I., Fatima A., Naz S. (2012). A p.C343S missense mutation in *PJVK* causes progressive hearing loss. Gene.

[18] Sloan-Heggen C.M., Babanejad M., Beheshtian M., Simpson A.C., Booth K.T., Ardalani F., Frees K.L., Mohseni M., Mozafari R., Mehrjoo Z. (2015). Characterising the spectrum of autosomal recessive hereditary hearing loss in Iran. J. Med. Genet..

[19] Zhang Q.J., Lan L., Li N., Qi Y., Zong L., Shi W., Yu L., Wang H., Yang J., Xie L.Y. (2015). Identification of a novel mutation of PJVK in the Chinese non-syndromic hearing loss population with low prevalence of the PJVK mutations. Acta Otolaryngol..

[20] Bademci G., Foster J., Mahdieh N., Bonyadi M., Duman D., Cengiz F.B., Menendez I., Diaz-Horta O., Shirkavand A., Zeinali S. (2016). Comprehensive analysis via exome sequencing uncovers genetic etiology in autosomal recessive nonsyndromic deafness in a large multiethnic cohort. Genet. Med..

[21] Yan D., Tekin D., Bademci G., Foster J., Cengiz F.B., Kannan-Sundhari A., Guo S., Mittal R., Zou B., Grati M. (2016). Spectrum of DNA variants for non-syndromic deafness in a large cohort from multiple continents. Hum. Genet..

[22] Alimardani M., Hosseini S.M., Khaniani M.S., Haghi M.R., Eslahi A., Farjami M., Chezgi J., Derakhshan S.M., Mojarrad M. (2019). Targeted Mutation Analysis of the SLC26A4, MYO6, PJVK and CDH23 Genes in Iranian Patients with AR Nonsyndromic Hearing Loss. Fetal Pediatr. Pathol..

[23] Khan A., Han S., Wang R., Ansar M., Ahmad W., Zhang X. (2019). Sequence variants in genes causing nonsyndromic hearing loss in a Pakistani cohort. Mol. Genet. Genomic Med..

[24] Richard E.M., Santos-Cortez R.L.P., Faridi R., Rehman A.U., Lee K., Shahzad M., Acharya A., Khan A.A., Imtiaz A., Chakchouk I. (2019). Global genetic insight contributed by consanguineous Pakistani families segregating hearing loss. Hum. Mutat..

[25] Zhou Y., Tariq M., He S., Abdullah U., Zhang J., Baig S.M. (2020). Whole exome sequencing identified mutations causing hearing loss in five consanguineous Pakistani families. BMC Med. Genet..

[26] Salime S., Charif M., Bousfiha A., Elrharchi S., Bakhchane A., Charoute H., Kabine M., Snoussi K., Lenaers G., Barakat A. (2017). Homozygous mutations in PJVK and MYO15A genes associated with non-syndromic hearing loss in Moroccan families. Int. J. Pediatr. Otorhinolaryngol..

[27] Starr A., Picton T.W., Sininger Y., Hood L.J., Berlin C.I. (1996). Auditory neuropathy. Brain.

[28] Arslan E., Genovese E., Orzan E., Turrini M. (1997). Valutazione della Percezione Verbale nel Bambino Ipoacusico.

[29] Elliott L.L., Katz D. (1980). Development of a New Children’s Test of Speech Discrimination (Technical Manual).

[30] Dib C., Fauré S., Fizames C., Samson D., Drouot N., Vignal A., Millasseau P., Marc S., Hazan J., Seboun E. (1996). A comprehensive genetic map of the human genome based on 5,264 microsatellites. Nature.

[31] Richards S., Aziz N., Bale S., Bick D., Das S., Gastier-Foster J., Grody W.W., Hegde M., Lyon E., Spector E. (2015). ACMG Laboratory Quality Assurance Committee. Standards and guidelines for the interpretation of sequence variants: A joint consensus recommendation of the American College of Medical Genetics and Genomics and the Association for Molecular Pathology. Genet. Med..

[32] VarSome: The Human Genomic Variant Search Engine. https://varsome.com/.

[33] Oza A.M., DiStefano M.T., Hemphill S.E., Cushman B.J., Grant A.R., Siegert R.K., Shen J., Chapin A., Boczek N.J., Schimmenti L.A. (2018). ClinGen Hearing Loss Clinical Domain Working Group. Expert specification of the ACMG/AMP variant interpretation guidelines for genetic hearing loss. Hum. Mutat..

[34] Genome Aggregation Database (gnomAD). https://gnomad.broadinstitute.org/.

[35] Boothroyd A., Madell J.R., Flexer C. (2008). The acoustic speech signal. Pediatric audiology: Diagnosis, technology, and management.

[36] Del Castillo I., Morín M., Domínguez-Ruiz M., Moreno-Pelayo M.A. (2022). Genetic Etiology of Non-Syndromic Hearing Loss in Europe. Hum. Genet..

